# Biomarkers of pulmonary hypertension in patients with scleroderma: a case–control study

**DOI:** 10.1186/s13075-015-0712-4

**Published:** 2015-08-06

**Authors:** Zsuzsanna McMahan, Florian Schoenhoff, Jennifer E. Van Eyk, Fredrick M. Wigley, Laura K. Hummers

**Affiliations:** Division of Rheumatology, Department of Medicine, Johns Hopkins University, 5200 Eastern Avenue, Mason F. Lord Building, Center Tower, Suite 4000, Baltimore, MD 21224 USA; Department of Cardiovascular Surgery, University Hospital Berne, Berne, Switzerland; Department of Medicine, School of Medicine, Johns Hopkins University, Baltimore, MD USA; Department of Biomedical Engineering, School of Medicine, Johns Hopkins University, Baltimore, MD USA; Department of Biological Chemistry, School of Medicine, Johns Hopkins University, Baltimore, MD USA; Present address: Advanced Clinical Biosystems Research Institute, Barbra Streisand Women’s Heart Center, Cedars-Sinai Heart Institute, Cedar-Sinai Medical Center, Los Angeles, CA 90048 USA

## Abstract

**Introduction:**

Significant pulmonary vascular disease is a leading cause of death in patients with scleroderma, and early detection and early medical intervention are important, as they may delay disease progression and improve survival and quality of life. Although several biomarkers have been proposed, there remains a need to define a reliable biomarker of early pulmonary vascular disease and subsequent development of pulmonary hypertension (PH). The purpose of this study was to define potential biomarkers for clinically significant pulmonary vascular disease in patients with scleroderma.

**Methods:**

The circulating growth factors basic fibroblast growth factor, placental growth factor (PlGF), vascular endothelial growth factor (VEGF), hepatocyte growth factor, and soluble VEGF receptor 1 (sFlt-1), as well as cytokines (interleukin [IL]-1β IL-2, IL-4, IL-5, IL-8, IL-10, IL-12, IL-13, tumor necrosis factor-α, and interferon-γ), were quantified in patients with scleroderma with PH (*n* = 37) or without PH (*n* = 40). In non-parametric unadjusted analyses, we examined associations of growth factor and cytokine levels with PH. In a subset of each group, a second set of earlier samples, drawn 3.0±1.6 years earlier, were assessed to determine the changes over time.

**Results:**

sFlt-1 (*p* = 0.02) and PlGF (*p* = 0.02) were higher in the PH than in the non-PH group. sFlt-1 (ρ = 0.3245; *p* = 0.01) positively correlated with right ventricular systolic pressure. Both PlGF (*p* = 0.03) and sFlt-1 (*p* = 0.04) positively correlated with the ratio of forced vital capacity to diffusing capacity for carbon monoxide (DLCO), and both inversely correlated with DLCO (*p* = 0.01). Both PlGF and sFlt-1 levels were stable over time in the control population.

**Conclusions:**

Our study demonstrated clear associations between regulators of angiogenesis (sFlt-1 and PlGF) and measures of PH in scleroderma and that these growth factors are potential biomarkers for PH in patients with scleroderma. Larger longitudinal studies are required for validation of our results.

**Electronic supplementary material:**

The online version of this article (doi:10.1186/s13075-015-0712-4) contains supplementary material, which is available to authorized users.

## Introduction

Pulmonary hypertension (PH) is a common complication of scleroderma, with an estimated 8–12 % of patients developing pulmonary arterial disease [8, 19]. Significant pulmonary vascular disease is a leading cause of death in patients with scleroderma, with approximately 30 % of scleroderma-related deaths being attributable to PH [24]. Early detection of pulmonary arterial hypertension (PAH) in patients with scleroderma, together with early medical interventions, is important, as it may provide the opportunity to delay disease progression and improve survival and quality of life [7, 8, 11, 22]. As targeted therapies for PH and PAH in scleroderma improve, early detection and intervention will become increasingly important.

Circulating biomarkers have the potential of playing a significant clin ical role in defining disease activity and predicting prognosis. Although several biomarkers have been proposed [1, 2, 6], there remains a need to define a reliable biomarker of early pulmonary vascular disease and provide insight into disease mechanisms. The goal of this pilot study was to investigate potentially relevant biomarkers associated with the presence of PH that could ultimately be tested in a longitudinal cohort study.

## Methods

### Subjects

We selected subjects from the Johns Hopkins Scleroderma Center who either met the 1980 American College of Rheumatology criteria for the diagnosis of scleroderma or had at least three of five features of CREST syndrome (calcinosis, Raynaud’s phenomenon, esophageal dysmotility, sclerodactyly, and/or telangiectasia). Additional selection criteria included the availability of two plasma samples drawn at least 6 months apart and data defining the presence or supporting the absence of PH. Patients were scored by clinicians at routine clinical visits as having or not having PH based on available data (“yes” or “no” on the clinical research form). Patients seen between 2005 and 2009 and documented as having PH were then further screened for inclusion in the study. Verification of the diagnosis of PH was done through review of right heart catheterization (RHC) data, data obtained from echocardiograms (echo), and chart reviews. Patients were defined as having PH if they had a resting RHC mean pulmonary arterial pressure (mPAP) ≥25 mmHg. Additional subgroups included the following groups according to World Health Organization (WHO) PH guidelines: (1) PAH with mPAP ≥25 mmHg, pulmonary capillary wedge pressure (PCWP) ≤15 mmHg, and forced vital capacity (FVC) ≥70 %; (2) PH and significant restrictive ventilatory defects (RVD) (mPAP ≥25 mmHg, PCWP <15 mmHg, and FVC <70 %); and (3) pulmonary venous hypertension (PVH) (mPAP ≥25 mmHg, PCWP >15 mmHg). Control subjects had no clinical symptoms suggestive of PH and had a diffusing capacity for carbon monoxide (DLCO) >60 %, an echo demonstrating right ventricular systolic pressure (RVSP) <45 mmHg, normal right atrial size (<16 cm^2^), and normal right ventricular function [5].

All patients and controls were followed at the Johns Hopkins Scleroderma Center, where clinical and laboratory data are collected prospectively every 6 months. Clinical data were selected at the time closest to the blood sample and included Medsger severity scores [20], demographics, pulmonary function tests, echocardiograms, and RHCs. FVC and DLCO were standardized for age and sex and reported as the percentage of the predicted value [9, 14]. A disproportionately low DLCO was defined by a ratio of forced vital capacity to diffusing capacity for carbon monoxide (FVC/DLCO) ≥1.6. The study was approved by the Johns Hopkins University’s Institutional Review Board, and all enrolled patients signed informed consent forms.

### Samples

Plasma samples in the scleroderma PH group (cases; *n* = 37) were drawn (in sodium citrate) after the diagnosis of PH was established. In the non-PH group (controls; *n* = 40), the most recent samples available were selected for analysis. In addition, a second, earlier plasma sample was selected from a subset of patients in the PH group (*n* = 12) and the non-PH group (*n* = 21) who had an available sample drawn at least 6 months before the first sample (cases, mean±standard deviation [SD] of 3.9±1.7 years before; controls, 2.5±1.4 years before) and before the diagnosis of PH in the case group. These samples were included to evaluate the variability of factor levels over time. Plasma samples were stored at −80 °C from collection until the time of analysis.

Samples were thawed and centrifuged with 15,000 relative centrifugal force for 15 minutes immediately before use. The growth factor levels (basic fibroblast growth factor [bFGF], placental growth factor [PlGF], vascular endothelial growth factor [VEGF], hepatocyte growth factor [HGF], VEGF receptor 1 [sFlt-1]) and cytokines (interleukin [IL]-1β, IL-2, IL-4, IL-5, IL-8, IL-10, IL-12, IL-13, tumor necrosis factor [TNF]-α, interferon [IFN]-γ) were measured using a ruthenium-based electrochemiluminescence platform (Meso Scale Discovery, Gaithersburg, MD, USA). Biomarkers were chosen based on preliminary or published data suggesting a possible biological association with PH and/or scleroderma-associated PH [3, 12, 13].

Duplicate samples were run using 25 μl of plasma per well. The results were considered valid when the percentage recovery was 100±20 %, the percentage of coefficient of variation (CV) was <20 %, the intra-assay CV was <10 %, and the interassay CV was <20 % and if 85 % of the samples of a run met these specifications. Each 96-well plate included standard curves that were used to determine concentrations and the lower limits of detection (LLOD) and quantification (LLOQ) [15, 18].

### Statistical analysis

Biomarkers with >25 % of their values outside the LLOQ of the assay were not analyzed further owing to sample size limitations. Differences in baseline characteristics between patients with PH (*n* = 37) and those without PH (*n* = 40) were examined with χ^2^ tests. Student’s *t* tests were used to compare the differences in means of normally distributed continuous variables in patients with and without PH. The Wilcoxon-Mann–Whitney test was used to compare the distribution of non-parametric continuous variables (including biomarkers) in patients with and without PH. This test was also used to define the association between biomarkers and other dichotomous demographic characteristics, including sex, race, cutaneous subtype, antibody status (anti-centromere protein [anti-CENP], anti-topoisomerase-1 [anti-Topo], and anti-ribonucleoprotein [anti-RNP]), and history of smoking. The relationship between the biomarker levels and other continuous variables, including age, disease duration (both from Raynaud’s and non-Raynaud’s symptom onset), DLCO, FVC/DLCO ratio, RVSP, mPAP, and the patient’s maximum Raynaud’s Medsger severity scores were examined with the Spearman correlation test. Limited multivariable regression modeling was done to evaluate the adjusted association between PH and the biomarkers of interest. Dependent variables included in the model were variables that were potentially relevant confounders in the univariate analyses.

The Wilcoxon-Mann–Whitney test was also used to evaluate differences in biomarker levels in patients with and without PH at the early time point to determine if differences in biomarker levels before the diagnosis of PH are informative. The difference in biomarker levels before and after the diagnosis of PH was evaluated using the non-parametric sign test. The stability of the biomarkers over time was evaluated by comparing biomarker levels among controls across the two time points with the non-parametric sign test. A subgroup analysis using analysis of variance was done to evaluate differences in biomarker levels in PAH, PVH, and PH-RVD. The data were analyzed using STATA version 11 statistical software (StataCorp, College Station, TX, USA).

## Results

### Characteristics of the patient population

The dataset comprised 77 patients: 37 patients with PH and 40 control subjects without PH (Table 1). The mean ages at sample collection were 65 (range, 38–89) years in the PH group and 56 (range, 27–73) years in the non-PH group (*p* = 0.001). As PH is more likely to present with increasing age [20], the age difference was expected. The median disease durations from the time of sample collection were 10.7 (interquartile range [IQR], 1.4–16.0) years in the PH group and 8.2 (IQR, 3.8–9.7) years in the non-PH group (*p* = 0.02). Differences related to sex (*p* = 1.00) and scleroderma subtype (*p* = 0.06) were not significant. Patients with PH were more frequently African American than those without PH (19.0 % vs. 2.5 %; *p* = 0.03). Twenty-eight of 37 patients with PH were receiving treatment for PH at the time of the study (Additional file 1: Table S1). WHO functional classes represented in the PH population included class 1 (*n* = 13), class 2 (*n* = 10), class 3 (*n* = 6), and class 4 (*n* = 8). Of the PH patients, 54 % (*n* = 20) had significant RVD, whereas significant RVD was present in only 7.5 % (*n* = 3) of those without PH (*p* < 0.001). Sixteen patients had PAH without significant RVD, 13 had PH and significant RVD, 8 patients had PVH (mPAP ≥25 mmHg, PCWP >15 mmHg), 5 of whom had significant RVD (FVC <70% of predicted).

**Table 1:** Baseline demographic and clinical characteristics of the entire study population

	Pulmonary hypertension (*n* = 37)	Controls (*n* = 40)	*p* value
Sex, *n* (%)			1.00
Male	3 (8.1)	4 (10.0)	
Female	34 (91.9)	36 (90.0)	
Mean age at last sample, yr (range)	64.9 (38–89)	55.9 (27–73)	<0.01
Race, *n* (%)			0.03
White	30 (81.1)	39 (97.5)	
African American	7 (18.9)	1 (2.5)	
Median disease duration, yr (IQR)^a^	10.7 (1.4–16.0)	8.2 (3.8–9.7)	<0.01
Limited SSc, *n* (%)	31 (83.8)	26 (65)	0.06
Ever smoker, *n* (%)	12 (32.4)	15 (37.5)	0.64
Median modified Rodnan skin score (0–51) (IQR)	7.0 (5.0–15.0)	7.0 (4.5–18.0)	0.97
Severe Raynaud’s symptoms, *n* (%)^b^	29 (78)	16 (40)	<0.01
FVC, mean±SD	67.5±21.2	88.1±15.9	<0.01
DLCO, mean±SD	48.4±18.9 (*n* = 36)	92.1±24.0	<0.01
Median RVSP (IQR)	60 (48–74.5) (*n* = 36)	33 (27–36) (*n* = 25)	<0.01
Anti-Scl-70, *n* (%)	5 (13.5)	7 (17.5)	0.63
Anti-RNP, *n* (%)	4 (11.4) (4/35)	2 (5.9) (2/34)	0.67
Anti-centromere, *n* (%)	17 (46.0)	13 (32.5)	0.23

*Abbreviations*: *DLCO* diffusing capacity of carbon monoxide, *FVC* forced vital capacity, *IQR* interquartile range, *RNP* ribonucleoprotein, *RVSP* right ventricular systolic pressure, *Scl*-*70* topoisomerase-1, *SD* standard deviation, *SSc* scleroderma

Non-parametric continuous data were evaluated using the Wilcoxon-Mann–Whitney test and are represented as median (IQR)

^a^From time of Raynaud’s onset

^b^Based on Medsger severity score (Medsger score >1 = severe Raynaud’s)

### Evaluating the association of biomarkers with clinical and demographic variables and the presence of pulmonary hypertension across cases and controls

We eliminated IL-1β, IFN-γ, IL-2, IL-4, IL-10, and IL-13 from the analysis, given that more than 25 % (27–65 %) of their enzyme-linked immunosorbent assay values were out of the quantifiable range of the assay (mainly below the LLOD). We found no associations between the biomarkers and sex, race, cutaneous subtype, antibody status (anti-CENP, anti-Topo, anti-RNP), or history of smoking. We also evaluated the association between biomarker levels and the continuous variables age, maximum Rodnan skin score (range, 0–51), and disease duration. We found that IL-8 (*p* = 0.02), TNF-α (*p* = 0.02), sFlt-1 (*p* = 0.02), and HGF (*p* = 0.01) were associated with age and that there were no significant associations between biomarkers and skin score or disease duration.

**Fig. 1:**
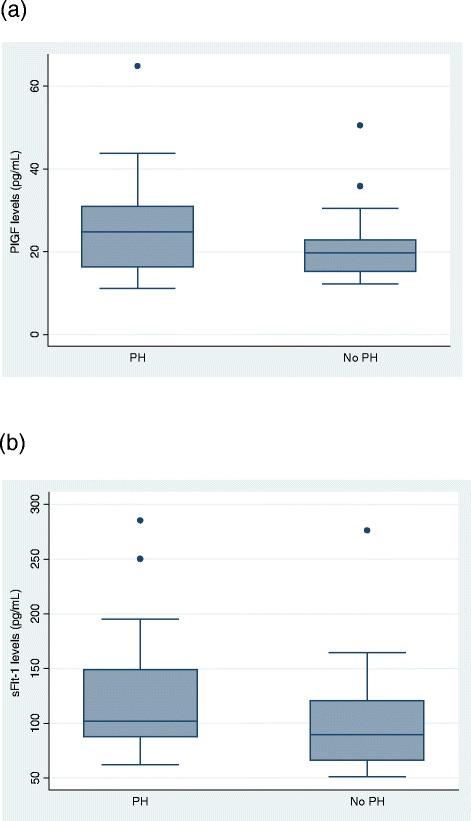
Placental growth factor (PlGF) and soluble fms-like tyrosine kinase-1 (sFlt-1) levels in patients with or without pulmonary hypertension (PH). **a** PlGF (median, 25 [interquartile range {IQR}, 16.3–31.0] pg/ml vs. 19.1 [15.3–22.9] pg/ml; *p* = 0.02) is significantly higher in patients with scleroderma with PH than in those with scleroderma without PH across the whole cohort at the later time point (*n* = 33 PH, *n* = 38 non-PH) (**b**) sFlt-1 (median, 102 [IQR, 88–149] pg/ml vs. 90 [66–121] pg/ml; *p* = 0.02) is significantly higher in patients with scleroderma with PH than in those with scleroderma without PH across the whole cohort (*n* = 37 PH, *n* = 40 non-PH)

We then evaluated the association between PH and each of the measurable biomarkers at the late time point and found that sFlt-1 (median, 101.8 [interquartile range {IQR}, 87.6–149.2] pg/ml vs. 89.7 [65.9–120.7] pg/ml; *p* = 0.02) and PlGF (median, 24.8 [IQR, 16.3–31.0] pg/ml vs. 19.1 [15.3–22.9] pg/ml; *p* = 0.02) are both significantly higher in patients with PH than in those without PH (Fig. 1a, b). None of the other biomarkers were associated with PH (Table 2).

**Table 2:** Biomarker levels in all patients with or without pulmonary hypertension with scleroderma

Biomarkers	PH (*n* = 37)	No PH (*n* = 40)	*p* value
IL-5	1.0 (0.7–1.6) (*n* = 35)	1.4 (0.8–2.0) (*n* = 35)	0.65
IL-8	7.2 (3.9–30.4)	5.7 (4.0–108.4) (*n* = 39)	0.95
IL-12	2.0 (1.3–4.3) (*n* = 35)	2.5 (1.5–4.5) (*n* = 38)	0.44
TNF-α	9.2 (6.2–18.8)	6.5 (4.4–113.9)	0.53
VEGF	174.0 (101.6–317.3)	129.0 (75.6–446.7)	0.51
bFGF	11.0 (5.5–21.3) (*n* = 33)	8.1 (4.0–19.3) (*n* = 36)	0.52
PlGF	24.8 (16.3–31.0) (*n* = 33)	19.7 (15.3–22.9) (*n* = 33)	0.02
sFlt-1	101.8 (87.6–149.2)	89.7 (65.9–120.7)	0.02
HGF	241.1 (153.3–400.5)	181.6 (101.6–391.1)	0.17

*Abbreviations*: *bFGF* basic fibroblast growth factor, *HGF* hepatocyte growth factor, *IL* interleukin, PH pulmonary hypertension, *PlGF* placental growth factor, *sFlt*-*1* soluble fms-like tyrosine kinase-1, *TNF* tumor necrosis factor, *VEGF* vascular endothelial growth factor

Data are presented as median (interquartile range) in picograms per milliliter.

Numbers vary between cytokines and growth factors because of slight variations in the number of values that were excluded from each group (those values that were out of quantifiable range of the assay)

We then performed limited multivariable modeling to evaluate the adjusted association between PlGF and sFlt-1 levels and scleroderma-associated PH. We adjusted for age and severe RVD in our model because they were potential confounders in the univariate analysis. In these models, the association between the biomarkers and PH was not significant, though the power to detect this association was limited by the small sample size.

To evaluate PH subgroups (PAH, PAH-RVD, and PVH), for differences in individual biomarker levels, a sensitivity analysis was done. Biomarker levels were not significantly different among the PH subtypes. In addition, biomarker levels were not different based on whether a patient was taking a prescribed vasodilator for PH (prostacyclin/prostaglandins, phosphodiesterase inhibitors, or endothelial receptor antagonists). The distribution of these medications across our patient population is listed in Additional file 2: Table S2.

### Evaluating biomarkers and measures of cardiopulmonary severity

**Fig. 2:**
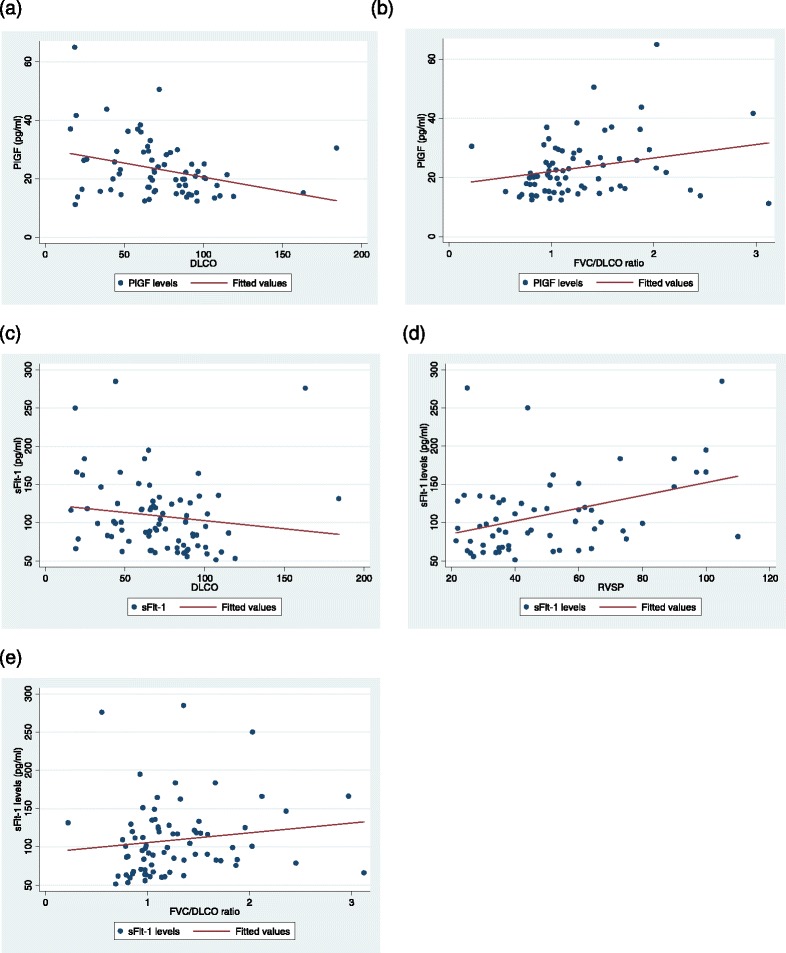
Placental growth factor (PlGF) and soluble fms-like tyrosine kinase-1 (sFlt-1) levels associate with measures of cardiopulmonary severity in scleroderma. **a** and **b** PlGF levels inversely correlate with diffusing capacity for carbon monoxide (DLCO) (ρ = −0.31; *p* = 0.01; *n* = 70) and positively correlate with forced vital capacity (FVC)/DLCO ratio (ρ = 0.26; *p* = 0.03; *n* = 70). **c**–**e** sFlt-1 levels inversely correlate with DLCO (ρ = −0.29; *p* = 0.01; *n* = 76) and positively correlate with right ventricular systolic pressure (RVSP) (ρ = 0.32; *p* = 0.01; *n* = 61) and FVC/DLCO ratio (ρ = 0.23; *p* = 0.04; *n* = 76). Low DLCO, high RVSP, and high FVC/DLCO ratio are measures of pulmonary vascular disease

Spearman’s correlation was then used to determine if there was a relationship between the biomarker levels and clinical indications of pulmonary disease severity (DLCO, FVC/DLCO ratio, and RVSP) across cases and controls. DLCO values were available for 76 of 77 patients. Interestingly, both PlGF (ρ = −0.31, *p* = 0.01) and sFlt-1 (ρ = −0.29, *p* = 0.01) were inversely correlated with DLCO and were significantly associated with the FVC/DLCO ratio (PlGF, ρ = 0.26, *p* = 0.03; sFlt-1, ρ = 0.23, *p* = 0.04) (Fig. 2). None of the other biomarkers were associated with these parameters. Both sFlt-1 (ρ = 0.32, *p* = 0.01) and HGF (ρ = 0.34, *p* = 0.01) were associated positively with RVSP, which was not the case for the other biomarkers.

### Evaluating biomarker levels and measures of peripheral vascular severity

**Fig. 3:**
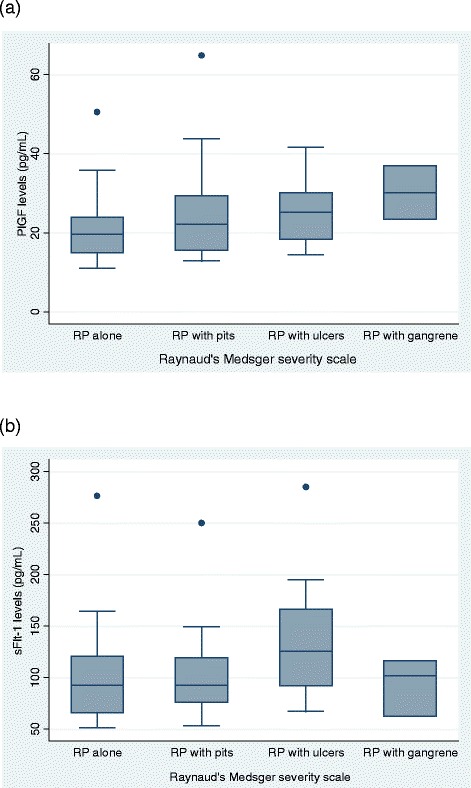
Placental growth factor (PlGF) levels are associated with severity of peripheral vascular manifestations in scleroderma. **a** PlGF levels correlate with Raynaud’s phenomenon (RP) severity as measured with the Raynaud’s Medsger severity scale (ρ = 0.29, *p* = 0.02; *n* = 71). **b** Soluble fms-like tyrosine kinase-1 (sFlt-1) levels correlate with Raynaud’s severity (ρ = 0.23, *p* = 0.04; *n* = 77)

We then explored whether biomarker levels correlate with clinical indications of peripheral vascular disease in scleroderma (*n* = 77). First, we correlated biomarker levels with measures of Raynaud’s severity in scleroderma [17, 23]. Interestingly, we found that levels of PlGF significantly correlated with Raynaud’s severity scores (ρ = 0.29, *p* = 0.02) (Fig. 3a). sFlt-1 levels also significantly correlated with Raynaud’s severity scores (ρ = 0.23, *p* = 0.04) (Fig. 3b). These associations were not observed with any of the other cytokines or growth factors we studied.

### Assessment of serial biomarker levels

In the patients who had two plasma samples available, with the first drawn before the diagnosis of PH (cases drawn, mean±SD of 3.9±1.7 years before late blood sample; controls drawn 2.5±1.4 years before late blood sample), biomarker levels from the prediagnosis sample (*n* = 12) were evaluated. These analyses were done to determine if patients who were eventually diagnosed with PH (*n* = 12) had biomarker levels at the early time point significantly different from those of patients who did not develop PH (*n* = 21). In the PH group at the early time point, levels of sFlt-1 (*p* = 0.02) and IL-12 (*p* = 0.03), but not any other markers, were significantly elevated relative to the non-PH group (Table 3). Levels of PlGF were also high in the PH group at the early time point, a finding that neared statistical significance (*p* = 0.07). At the early time point, PlGF levels positively correlated with RVSP (ρ = 0.54; *p* < 0.01; *n* = 25) and inversely associated with DLCO (ρ = −0.35; *p* = 0.06). The inverse association between sFlt-1 and DLCO (ρ = −0.34; *p* = 0.05) and the positive correlation with RVSP (ρ = 0.35; *p* = 0.074) both neared statistical significance.

**Table 3:** Association of biomarker levels at the early time point with the later diagnosis of pulmonary hypertension

Biomarker	PH (*n* = 12)	No PH (*n* = 21)	*p* value
IL-5	1.1 (1.0–1.7)	1.0 (0.8–1.7)	0.38
IL-8	17.5 (6.01–153.2)	7.6 (5.2–19.4)	0.20
IL-12	2.9 (2.0–8.2)	1.8 (1.5–3.3)	0.03
TNF-α	7.2 (4.6–22.3)	5.9 (4.3–10.0)	0.50
VEGF	386.7 (183.1–661.6)	388.81 (199.0–498.2)	0.91
bFGF	16.8 (7.1–42.7)	11.8 (6.3–19.5)	0.41
PlGF	24.9 (20.9–26.8)	19.3 (15.1–24.8)	0.07
sFlt-1	124.0 (101.5–138.5)	99.2 (84.2–113.0)	0.02
HGF	249.0 (148.1–568.0)	176.1 (139.2–253.9)	0.18

*Abbreviations*: *bFGF* basic fibroblast growth factor, *HGF* hepatocyte growth factor, *IL* interleukin, PH pulmonary hypertension, *PlGF* placental growth factor, *sFlt*-*1* soluble fms-like tyrosine kinase-1, *TNF* tumor necrosis factor, *VEGF* vascular endothelial growth factor

Data are presented as median (interquartile range) in picograms per milliliter

Interestingly, FVC/DLCO ratios (ρ = 0.53; *p* < 0.01) were positively associated with bFGF, inversely associated with DLCO (ρ = −0.356; *p* = 0.046), and associated with high bFGF levels. HGF also was inversely associated with DLCO (ρ = −0.33; *p* = 0.06).

### Evaluating stability of biomarkers over time in controls

We then assessed the stability of serial biomarkers over time in the control group. We found that all biomarkers, except for VEGF (*p* = 0.03), were stable over time, with no significant difference in levels between the early and late time points (Additional file 2: Table S2).

## Discussion

The major finding in our study was that clear associations exist between regulators of angiogenesis (sFlt-1 and PlGF) and measures of pulmonary vascular disease and that these growth factors are potential biomarkers for scleroderma-related PH. Both sFlt-1 and PlGF were associated with PH and clinical markers of vascular disease. sFlt-1 levels were positively correlated with FVC/DLCO ratio and RVSP and inversely correlated with DLCO, and PlGF levels were also positively correlated with FVC/DLCO and inversely correlated with DLCO. These patterns were not evident for the other biomarkers. In addition, both PlGF and sFlt-1 were significantly associated with Raynaud’s severity scores. Levels of both PlGF and sFlt-1 were stable over time in the control population.

Interestingly, PlGF was recently studied as a predictor of vascular complications related to Raynaud’s phenomenon in scleroderma [3]. This study demonstrated that elevated PlGF levels in scleroderma predict new digital ulcers, highlighting a potential role of PlGF in scleroderma-related vascular disease [3]. In a prior study done at our center, investigators evaluated PlGF and other factors in scleroderma as potentially relevant biomarkers for vascular disease in scleroderma. In that cross-sectional study of 113 patients with scleroderma, PlGF levels were markedly higher in patients with scleroderma than in control subjects (*p* < 0.0001) and were associated with echo-based estimates of RVSP levels (*p* = 0.037). These data are consistent with those in our current survey, supporting the association of circulating PlGF and pulmonary vascular disease.

sFlt-1 is a tyrosine kinase inhibitor that is released from endothelial cells and monocytes. sFlt-1 acts through the circulation to disrupt the interaction of PlGF and VEGF with endothelial cell surface receptors [16]. The endothelial cell response to VEGF is enhanced by high circulating levels of PlGF in many disorders [4], and VEGF and PlGF are required for endothelial cell homeostasis [24]. Endothelial cell dysfunction is thought to play a major role in the development of PH. In theory, PAH may be a consequence of an endothelial cell influence on vascular smooth muscle, either by releasing factors that stimulate smooth muscle proliferation or by failing to release relevant inhibitory factors [21]. Evidence also suggests that endothelial injury and/or dysfunction also plays a role in the development of PH associated with interstitial lung disease (ILD) and PVH [12].

Although the classifications of PH within scleroderma are currently divided between PAH, PH associated with lung disease, and PH associated with left heart disease, with currently available testing, we cannot exclude the presence of PAH in patients with existing lung and left heart disease in scleroderma, and there is no evidence that the affected biological pathways within these subgroups in scleroderma are entirely distinct. For example, when fitting patients with scleroderma into the current PH classification system, we assume that the presence of ILD excludes the presence of PAH, but this may not be true, as the two may coexist in the context of a common underlying disease process (scleroderma). Such clearly demarcated distinctions between these subsets of patients may be somewhat artificial, as there is likely overlap between PH groups. The lack of a difference in levels of sFlt-1 and PlGF among distinct PH subsets supports the idea that there may be some common disease mechanisms across the scleroderma PH subsets. Further confirmatory studies to evaluate this question are needed.

There are several potential limitations to our study. It remains unclear whether the elevated biomarkers levels are specific for scleroderma pulmonary vascular disease or if they are influenced by other scleroderma features, such as peripheral vascular disease or active tissue injury with fibrosis. We also did not control for the possible influence of medications. Second, this was an unmatched study, and comparison groups were slightly different at baseline, with the PH group being slightly older and having more significant RVD.

Because this was a pilot study meant to identify potentially useful biomarkers in a small group of patients, we did not correct for multiple comparisons or pursue extensive multivariable analyses, as this is best addressed in larger longitudinal studies. Despite our small population, the same two biomarkers (sFlt-1 and PlGF) were consistently associated with PH and its associated clinical measures, suggesting that this was not simply due to a type II error. Assessing biomarker levels at only two points in time is a limitation, as some biomarker levels can be highly variable. These levels will need to be assessed at multiple time points in a follow-up longitudinal cohort study. We do recognize that a prediagnosis sample may not be predisease; however, this evaluation is still relevant in a population of undiagnosed pre-PH patients, as they are evolving along a biological continuum, and these findings may provide some insight into disease mechanisms. Finally, we did not include a non-scleroderma control group, because our focus was scleroderma-related pulmonary vascular disease.

## Conclusions

Our survey provides compelling evidence that sFlt-1 and PlGF levels are associated with measures of pulmonary vascular disease and that these growth factors may be potential biomarkers for scleroderma PH. The association of these factors and PH also suggest that they may play a biological role in the pathogenesis of PH. This survey supports the need for larger, prospective, randomized studies to extend these preliminary observations.

## Additional files

Additional file 1: Table S1. Pulmonary hypertension medications in patients with and without PH. (PDF 8 kb) Additional file 2: Table S2. Biomarker stability over time in the control group. Table listing median biomarker levels at the two time points in the control group. The sign test is used to make the statistical comparisons between levels. (PDF 30 kb)

## Abbreviations

bFGF: Basic fibroblast growth factor
CENP: Centromere protein
CV: Coefficient of variation
DLCO: Diffusing capacity for carbon monoxide
echo: Echocardiogram
FVC: Forced vital capacity
FVC/DLCO: Ratio of forced vital capacity to diffusing capacity for carbon monoxide
HGF: Hepatocyte growth factor
IFN-γ: Interferon gamma
IL: Interleukin
ILD: Interstitial lung disease
IQR: Interquartile range
LLOD: Lower limit of detection
LLOQ: Lower limit of quantification
mPAP: Mean pulmonary arterial pressure
PAH: Pulmonary arterial hypertension
PCWP: Pulmonary capillary wedge pressure
PH: Pulmonary hypertension
PlGF: Placental growth factor
PVH: Pulmonary venous hypertension
RHC: Right heart catheterization
RNP: Ribonucleoprotein
RVD: Restrictive ventilator defect
RVSP: Right ventricular systolic pressure
sFlt-1: Soluble fms-like tyrosine kinase-1
TNF-α: Tumor necrosis factor alpha
Topo: Topoisomerase-1
VEGF: Vascular endothelial growth factor
WHO: World Health Organization
