# Supplementary material for: Biomarkers of pulmonary hypertension in patients with scleroderma: a case–control study
Source: Arthritis Res Ther. 2015 Aug 6;17(1):201. doi: 10.1186/s13075-015-0712-4 (PMC4527208; doi:10.1186/s13075-015-0712-4)
Supplement: Additional file 1: Table S1. — Pulmonary hypertension medications in patients with and without PH. (PDF 8 kb) [file 13075_2015_712_MOESM1_ESM.pdf]

**Additional Table 1. Pulmonary hypertension medications in patients with and without PH**

| | **PH (n=37)** | **No PH (n=40)** |
|---|---|---|
| Prostacyclin/prostaglandin, n (%) | 5 (13.5) | 0 (0) |
| Phosphodiesterase inhibitor, n (%) | 25 (67.6) | 3 (7.5) |
| Endothelial receptor antagonist, n (%) | 15 (40.5) | 0 (0) |

*PH = pulmonary hypertension
