# Supplementary material for: Biomarkers of pulmonary hypertension in patients with scleroderma: a case–control study
Source: Arthritis Res Ther. 2015 Aug 6;17(1):201. doi: 10.1186/s13075-015-0712-4 (PMC4527208; doi:10.1186/s13075-015-0712-4)
Supplement: Additional file 2: Table S2. — Biomarker stability over time in the control group. Table listing median biomarker levels at the two time points in the control group. The sign test is used to make the statistical comparisons between levels. (PDF 30 kb) [file 13075_2015_712_MOESM2_ESM.pdf]

Additional Table 2. Biomarker stability over time in the control group

Biomarker	Level in early control Median (IQR)	Level in late control Median (IQR)	p-value
IL-5	1.0 (0.8-1.7)	1.4 (0.8-23.5)	0.24
IL-8	7.6 (5.2-19.4)	4.9 (3.1-50.6)	0.26
IL-12	1.8 (1.5-3.3)	2.0 (1.4-4.5)	0.36
TNF-a	5.9 (4.3-10.0)	6.5 (4.7-95.2)	0.66
VEGF	388.8 (199.0-498.2)	110.2 (75.9-366.1)	**0.03**
BFGF	11.8 (6.3-19.5)	5.3 (3.7-17.7)	0.11
PlGF	19.3 (15.2-24.8)	19.9 (17.0-22.5)	1.00
s-Flt1	99.2 (84.2-113.0)	86.6 (67.2-128.3)	0.38
HGF	176.1 (139.2-253.9)	145.1 (82.9-269.2)	1.00

**Abbreviations: INF= interferon; TNF=tumor necrosis factor; IL= interleukin; bFGF= basic fibroblast growth factor; PLGF= placental growth factor; sFlt-1= soluble fms-like tyrosine kinase-1; VEGF= vascular endothelial growth factor; HGF= hepatocyte growth factor*
*****n's vary between cytokines/growth factors given slight variations in the number of values that were excluded from each group (those values which were out of quantifiable range of the assay)*
***the nonparametric sign test is used to compare early and late biomarker levels in controls*
